# FUNDC1-mediated mitophagy and HIF1α activation drives pulmonary hypertension during hypoxia

**DOI:** 10.1038/s41419-022-05091-2

**Published:** 2022-07-21

**Authors:** Ruxia Liu, Chunling Xu, Weilin Zhang, Yangpo Cao, Jingjing Ye, Bo Li, Shi Jia, Lin Weng, Yingying Liu, Lei Liu, Ming Zheng

**Affiliations:** 1 Ministry of Education Key Laboratory of Molecular Cardiovascular Science, Department of Physiology and Pathophysiology, School of Basic Medical Sciences, Peking University Health Science Center, Beijing, China; 2 Department of Rehabilitation, School of Medical Technology, Tianjin Medical University, Tianjin, China; 3 State Key Laboratory of Membrane Biology, Institute of Zoology, Chinese Academy of Sciences, Beijing, China

**Keywords:** Vascular diseases, Mitophagy

## Abstract

Hypoxic pulmonary hypertension (PH) is a progressive disease characterized by hyper-proliferation of pulmonary vascular cells including pulmonary artery smooth muscle cells (PASMCs) and can lead to right heart failure and early death. Selective degradation of mitochondria by mitophagy during hypoxia regulates mitochondrial functions in many cells, however, it is not clear if mitophagy is involved in the pathogenesis of hypoxic PH. By employing the hypoxic mitophagy receptor *Fundc1* knockout (KO) and transgenic (TG) mouse models, combined hypoxic PH models, the current study found that mitophagy is actively involved in hypoxic PH through regulating PASMC proliferation. In the pulmonary artery medium from hypoxic PH mice, mitophagy was upregulated, accompanied with the increased active form of FUNDC1 protein and the enhanced binding affinity of FUNDC1 with LC3B. In PASMCs, overexpression of FUNDC1 increased mitophagy and cell proliferation while knockdown of FUNDC1 inhibited hypoxia-induced mitophagy and PASMC proliferation. Stimulation of mitophagy by FUNDC1 in PASMCs elevated ROS production and inhibited ubiquitination of hypoxia inducible factor 1α (HIF1α), and inhibition of mitophagy by FUNDC1 knockdown or knockout abolished hypoxia-induced ROS-HIF1α upregulation. Moreover, *Fundc1* TG mice developed severe hemodynamics changes and pulmonary vascular remodeling, and *Fundc1* KO mice were much resistant to hypoxic PH. In addition, intraperitoneal injection of a specific FUNDC1 peptide inhibitor to block mitophagy ameliorated hypoxic PH. Our results reveal that during hypoxic PH, FUNDC1-mediated mitophagy is upregulated which activates ROS-HIF1α pathway and promotes PASMC proliferation, ultimately leads to pulmonary vascular remodeling and PH.

## Introduction

Pulmonary hypertension (PH) is a severe cardiopulmonary disease ultimately leading to right heart failure and early death [1]. Sustained vasoconstriction and vascular remodeling are hallmarks of PH. Pulmonary arterial remodeling is characterized by the thickness of the intimal/medial layer of vessels mainly resulting from hyper-proliferation, migration, and apoptosis-resistance of pulmonary arterial smooth muscle cells (PASMCs), causing functional stiffness of arteries and leading to pulmonary vascular hemodynamic changes [2]. The pathogenesis of PAH is extraordinary complicated, a variety of genetic and pathogenic factors has been identified to be associated with the development of PAH. Chronic hypoxia is the most intensively studied PH-inducing factor which causes pulmonary vascular remodeling especially in hypoxic PH [3]. Although alterations in molecules or signals such as increased Na^+^/H^+^ exchanger level and cellular pH, elevated intracellular calcium concentration, reduced potassium channel activity, increased Rho kinase activity and nuclear factor of activated T cells, and hypoxia inducible factor 1α (HIF1α) have been suggested to regulate hypoxia-induced PASMC proliferation and vascular remodeling [4–6], therapeutic interventions of PH with targeting these factors are not satisfactory. Thus, more studies to determine the mechanisms underlying hypoxia-induced PASMC proliferation with a focus on therapeutic strategies are required.

Autophagy is a conserved cellular process which occurs constitutively, helping to clear damaged organelles and cytotoxic protein aggregates to maintain cell homeostasis. Dysregulations of autophagy are associated with diverse diseases such as cancer [7], cardiac diseases [8], as well as vascular disease [9]. Recent studies have reported that autophagy is involved in the development of PH [10]. However, the functional roles of autophagy in PH are still controversial. For instance, mice with genetic deletion of autophagy marker LC3B or Beclin1 displayed more severe hypoxia-induced PH or persistent PH, while pharmacological inhibition of autophagy by autophagy inhibitor chloroquine improved rat PH [10–12]. In addition, activation of autophagy by rapamycin or analogs improved animal and human PH [13, 14]. Mitochondria are organelles where oxidative phosphorylation occurs to synthesize ATP through consumption of oxygen and where most reactive oxygen species (ROS) produce. A large body of evidence indicates that hypoxia induces mitochondrial ROS production, and ROS in turn causes induction of HIF and PH development [15, 16]. However, the role of mitophagy (selective degradation of mitochondria by autophagy) in PASMC proliferation and in hypoxic PH is not clear.

FUNDC1 is a hypoxia-induced mitophagy receptor which has a typical LC3-interacting region (LIR) in N-terminal to mediate mitophagy by directly interacting with LC3 [17, 18]. Hypoxia induces the dephosphorylation of FUNDC1 Tyr18 in LIR motif which has enhanced binding affinity with LC3, causing increased mitophagy [19, 20]. In the present study, we took advantage of FUNDC1 transgenic and knockout mouse models, combined with hypoxia-induced PH mouse model, to investigate the role of FUNDC1-mediated mitophagy in PASMC proliferation, pulmonary arterial remodeling, and hypoxic PH. Furthermore, we investigated the therapeutic possibility of targeted inhibition of mitophagy by a synthetic cell-penetrating peptide to intervene the development of PH.

## Results

### Mitophagy is upregulated in hypoxic PH

**Fig. 1:**
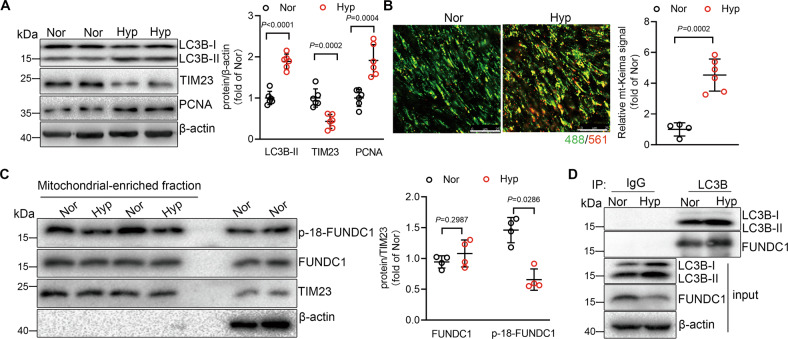
Hypoxia upregulates mitophagy in pulmonary arteries from pulmonary hypertension (PH) mice and in pulmonary artery smooth muscle cells (PASMCs). **A** Western blot of lung tissues isolated from normoxic (21% O_2_) or hypoxic mice (10% O_2_) using antibodies as indicated. *n* = 6 mice per group. **B** Confocal images of median layers of pulmonary arteries from mt-Keima mice treated with normoxia or hypoxia. Values are normalized to normoxic level of mitophagy. *n* = 4–6 mice per group, scale bar: 25 μm. **C** Western blot showing protein levels of p-18-FUNDC1 and FUNDC1 in mitochondria-enriched fraction from PASMCs under normoxia or hypoxia. *n* = 4 independent experiments per group. **D** Co-IP of LC3B with FUNDC1 in PASMCs with or without hypoxia treatment. Data are presented as mean ± SD. In **A**, **B**, data were analyzed using Mann–Whitney *U*-test. In **C**, *p* values were determined by one-way ANOVA followed by Bonferroni post hoc analysis.

Wild-type (WT) mice which were exposed to hypoxia for 3 weeks to induce hypoxic PH exhibited significantly increased right ventricular systolic pressure (RVSP), a surrogate for pulmonary arterial pressure (Supplementary Fig. S1A). The lung tissues showed increased LC3B lipidation, decreased mitochondrial membrane protein TIM23, and upregulated proliferating cell nuclear antigen (PCNA) (Fig. 1A), indicating increased autophagy including mitophagy and increased proliferation in response to hypoxia. mt-Keima is a pH-sensitive fluorescent protein targetedly expressed in mitochondria, which shows green fluorescence in the alkaline mitochondria and red in mitochondria engulfed in acid lysosome by mitophagy. We detected increased red fluorescent signal in pulmonary arteries from mt-Keima mice subjected to hypoxia for 3 weeks, as comparing with that from normoxic mice (Fig. 1B), suggesting the elevated mitophagy activity in hypoxic PH.

We next checked the mitophagy activity in PASMCs in response to hypoxia by measuring the hypoxia-induced mitophagy receptor FUNDC1 which contains a Y^18^EVL region in N-terminal and the dephosphorylation of Tyr18 is essential for FUNDC1-LC3 interaction [17, 18, 21]. In isolated mitochondria from rat PASMCs, hypoxia caused decreased level of phosphorylated FUNDC1 protein (Fig. 1C), indicating the increased mitophagy activity in response to hypoxia. Dephosphorylated FUNDC1 regulates mitophagy through interacting with LC3B-II protein, we then measured the interaction of FUNDC1 with LC3B-II in PASMCs with or without hypoxia treatment, and found that hypoxia caused increased interaction of FUNDC1 and LC3B-II (Fig. 1D), further indicating that hypoxia upregulates mitophagy activity through dephosphorylation of FUNDC1. Together, our results suggest that mitophagy is upregulated in hypoxic PH and in PASMCs in response to hypoxic stimulation.

### FUNDC1 causes proliferation of PASMCs in response to hypoxia

**Fig. 2:**
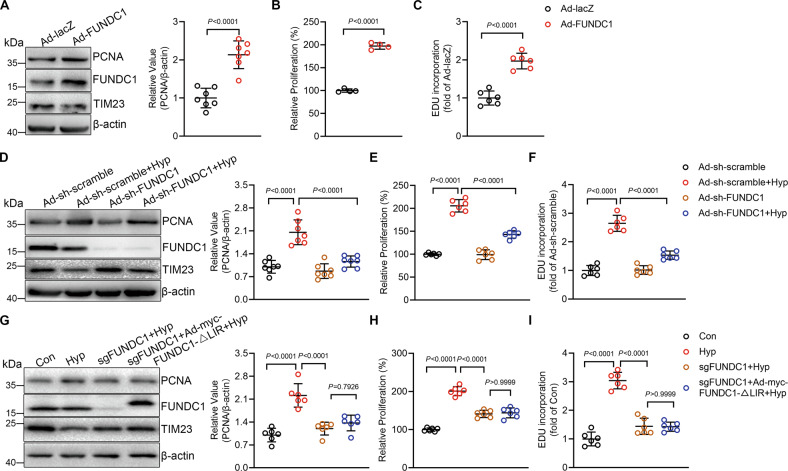
Increasing mitophagy induces and inhibition of mitophagy inhibits hypoxia-induced pulmonary artery smooth muscle cell (PASMC) proliferation. **A** Western blot showing proliferating cell nuclear antigen (PCNA) protein levels in Ad-lacZ and Ad-FUNDC1 PASMCs. *n* = 7 independent experiments per group. **B** CCK8 assay and **C** EDU assay of cells as in (**A**) *n* = 4 independent experiments per group for **B** and *n* = 6 independent experiments per group for (**C**). **D** Western blot showing PCNA levels in Ad-sh-scramble or Ad-sh-FUNDC1 PASMCs with or without hypoxia for 24 h. *n* = 7 independent experiments per group. **E** CCK8 assay and **F** EDU assay of cells as in **D**. *n* = 6 independent experiments per group for (**E**, **F**. **G**) Western blot showing PCNA levels in PASMCs by CRISPR/Cas-mediated knockout of FUNDC1 with or without stably expressing myc-FUNDC1-ΔLIR under hypoxia. *n* = 6 independent experiments per group. **H** CCK8 assay and **I** EDU assay of cells as in **G**. *n* = 6 independent experiments per group for (**H**, **I**). Data are presented as mean ± SD. Two-tailed unpaired Student’s *t* test and one-way ANOVA followed by Bonferroni post hoc analysis were used to compare two and multiple groups except for (**B**). In **B**, *p* values were determined by Mann–Whitney *U*-test.

Hyper-proliferation of vascular cells including PASMCs is an important feature of hypoxic PH [22]. We detected upregulated proliferation marker PCNA accompanied with increased autophagy in lung tissues from hypoxic PH (Fig. 1A), so we investigated if hypoxia-induced mitophagy mediates PASMC proliferation. We regulated mitophagy through adenovirus-mediated overexpressing or knockdown FUNDC1 (Ad-FUNDC1 or Ad-sh-FUNDC1) in PASMCs. Overexpression of FUNDC1 resulted in increased mitophagy as indicated by the increased interaction of FUNDC1 with LC3B (Supplementary Fig. S2A) and the red fluorescent signal of Keima (Supplementary Fig. S2B), and knockdown of FUNDC1 inhibited hypoxia-induced increase of mitophagy (Supplementary Fig. S2C). In PASMCs, overexpression of FUNDC1 caused increased PCNA protein level (Fig. 2A). In addition, upregulation of FUNDC1 also increased cell viability as detected by CCK8 assay and EDU incorporation (Fig. 2B, C), indicating that mitophagy increases cell proliferation. In contrast, while hypoxia induced PASMC proliferation as indicated by the increased PCNA protein level, cell viability and EDU incorporation (Fig. 2D–F), knockdown of FUNDC1 largely blocked hypoxia-mediated proliferation (Fig. 2D–F). Moreover, we generated FUNDC1 knockout PASMCs by CRISPR/Cas and re-expressed wildtype FUNDC1 or the FUNDC1-ΔLIR mutant lacking the LC3B-interacting region by adenovirus-mediated transfection (Supplementary Fig. S3, Fig. 2G). While FUNDC1 knockout PASMCs showed lower proliferation than WT PASMCs in response to hypoxia which could be reversed by re-expression of wildtype FUNDC1, re-expression of FUNDC1-ΔLIR could not reverse the effect (Supplementary Fig. S3, Fig. 2G–I). Together, these results confirm that the increased FUNDC1 activity caused by hypoxia regulates PASMC proliferation. In addition to cell proliferation, excessive PASMC migration and apoptotic resistance in the arterial wall are also dominant features leading to the vascular remodeling during PH [23]. Transwell assay and flow cytometry results showed that overexpression of FUNDC1 increased PASMC migration but reduced apoptosis (Supplementary Fig. S2D, E), and knockdown of FUNDC1 inhibited hypoxia-induced PASMC migration and apoptotic resistance (Supplementary Fig. S2F, G). Together, these results suggest that FUNDC1 is sufficient and necessary to PASMC proliferation during hypoxic PH.

### FUNDC1 stabilizes HIF1α to promote PASMC proliferation

**Fig. 3:**
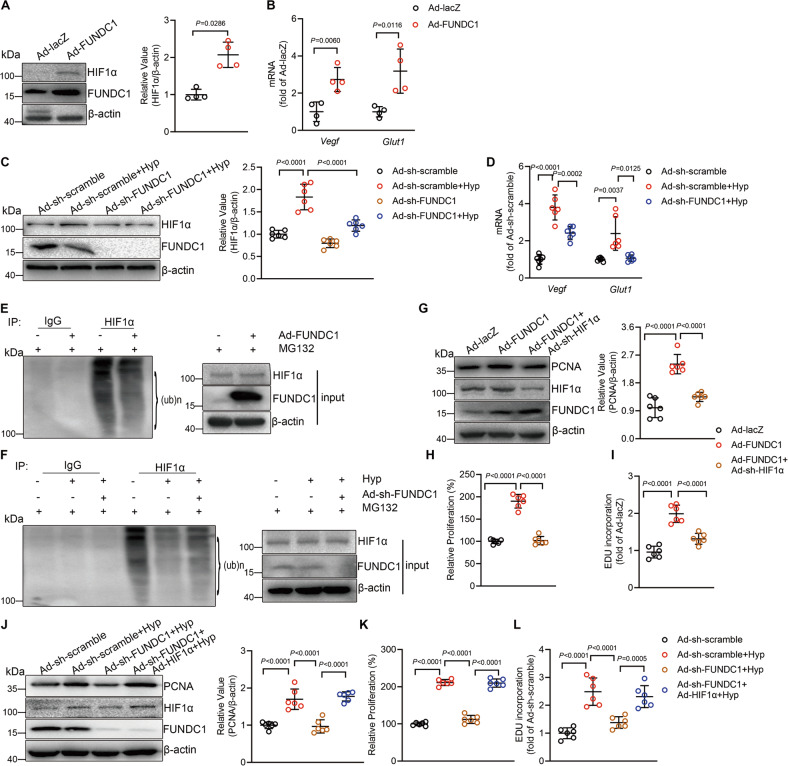
Mitophagy upregulates hypoxia inducible factor 1α (HIF1α) to induce pulmonary artery smooth muscle cell (PASMC) proliferation. **A** Western blot showing HIF1α levels in PASMCs transfected with Ad-lacZ or Ad-FUNDC1. *n* = 4 independent experiments per group, *p* values were determined by Mann–Whitney *U*-test. **B** mRNA levels of *Vegf* and *Glut1* in cells as in (**A**). *n* = 4 independent experiments per group, *p* values were determined by Mann–Whitney *U*-test. **C** HIF1α levels in PASMCs transfected with Ad-sh-scramble or Ad-sh-FUNDC1 under normoxia or hypoxia for 24 h. *n* = 6 independent experiments per group. **D** mRNA levels of *Vegf* and *Glut1* in cells as in (**C**). *n* = 6 independent experiments per group. *p* values were determined by one-way ANOVA followed by Bonferroni post hoc analysis for *Vegf* mRNA analysis, and Mann–Whitney *U*-test for *Glut1* mRNA analysis. **E** Ubiquitination assay with HIF1α showing the ubiquitination levels in PASMCs infected with Ad-lacZ or Ad-FUNDC1 in the presence of MG132. **F** The ubiquitination levels of HIF1α in Ad-sh-scramble and Ad-sh-FUNDC1 PASMCs in the presence of MG132 under hypoxia. **G** PCNA protein levels in PASMCs transfected with Ad-lacZ, Ad-FUNDC1 or Ad-FUNDC1 + Ad-sh-HIF1α. *n* = 6 independent experiments per group. **H** CCK8 assay and **I** EDU assay of cells as in (**G**). *n* = 6 independent experiments per group for each assay. **J** PCNA protein levels, **K** CCK8 assay, and **L** EDU assay of PASMCs with Ad-sh-FUNDC1 and Ad-HIF1α under hypoxia. *n* = 6 independent experiments per group for (**J**, **K**, **L**). Data are presented as mean ± SD. Two-tailed unpaired Student’s *t* test and one-way ANOVA followed by Bonferroni post hoc analysis were used to compare two and multiple groups except for (**A**, **B** and **D**) for *Glut1* mRNA analysis.

Next we seek to know how FUNDC1-mediated mitophagy regulates PASMC proliferation during hypoxic PH. It is known that mitochondria are organelles where oxidative phosphorylation occurs, and that hypoxia upregulates hypoxia inducible factor 1α (HIF1α) which in turn initiates transcription of multiple target genes including genes regulating PASMC proliferation [24]. We then checked if FUNDC1 regulates HIF1α in PASMCs during hypoxic PH. In PASMCs overexpressing FUNDC1 to induce mitophagy, HIF1α protein was markedly increased, as comparing with control cells (Fig. 3A). Consistently, mRNA levels of *Vegf* and *Glut1*, two target genes of HIF1α, also increased in response to the upregulation of mitophagy (Fig. 3B). Importantly, whereas hypoxia also increased HIF1α protein level and mRNA levels of *Vegf* and *Glut1*, the hypoxia-induced increase of HIF1α activity was suppressed by knockdown of FUNDC1 (Fig. 3C, D), indicating that hypoxia stimulates HIF1α activity through regulating mitophagy in PASMCs. HIF1α protein and activity are mainly regulated by ubiquitination [25], we then tested whether hypoxia-mediated FUNDC1 activity regulates the ubiquitination of HIF1α protein. Ubiquitination assay with HIF1α protein from FUNDC1 overexpressing or knockdown PASMCs in the presence of the proteasome inhibitor MG132 showed that FUNDC1 overexpression decreased the ubiquitination of HIF1α under normoxia, and FUNDC1 knockdown restored the suppressed HIF1α ubiquitination in response to hypoxia (Fig. 3E, F), indicating that FUNDC1 stabilizes HIF1α during hypoxia through inhibition of ubiquitination.

Next we investigated whether HIF1α activity is responsible for FUNDC1-caused PASMC proliferation. We first knocked down HIF1α in FUNDC1 overexpressing PASMCs. While knockdown of HIF1α did not change the increased FUNDC1 protein level in FUNDC1 overexpressing PASMCs, it largely suppressed the increased HIF1α protein level and PCNA protein level by FUNDC1 overexpression (Fig. 3G). Moreover, HIF1α knockdown inhibited FUNDC1-induced PASMC proliferation, as indicated by the CCK8 and EDU incorporation assays (Fig. 3H, I), suggesting that FUNDC1 mediates PASMC proliferation through HIF1α. Then, we overexpressed HIF1α in FUNDC1 knockdown PASMCs. HIF1α overexpression did not alter FUNDC1 protein level in FUNDC1 knockdown cells treated with hypoxia but it restored the FUNDC1 knockdown-suppressed elevated HIF1α protein level and PCNA protein level by hypoxia (Fig. 3J). Likewise, overexpressing HIF1α in FUNDC1 knockdown PASMCs rescued the hypoxia-induced proliferation which was blocked by FUNDC1 knockdown (Fig. 3K, L), indicating that HIF1α mediates hypoxia/ FUNDC1-induced PASMC proliferation. Together, our results suggest that hypoxia-induced FUNDC1 activity stimulates PASMC proliferation through regulating HIF1α stability.

### FUNDC1 induces PASMC proliferation through upregulating ROS-HIF1α pathway

**Fig. 4:**
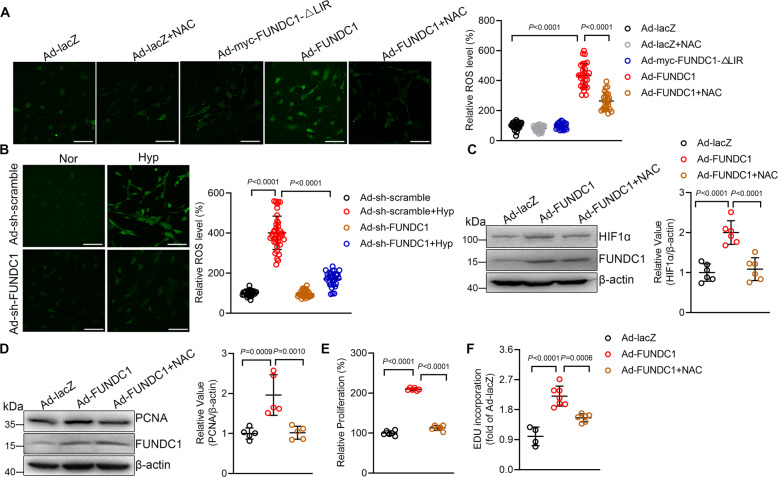
Mitophagy increases reactive oxygen species (ROS) and hypoxia inducible factor 1α (HIF1α) to induce pulmonary artery smooth muscle cell (PASMC) proliferation. **A** Confocal images showing ROS production in PASMCs with or without NAC (10 mM), as indicated by DCFH-DA fluorescent signal. *n* = 24–28 cells per group, scale bar:100 μm. **B** Confocal images of DCFH-DA in Ad-sh-scramble and Ad-sh-FUNDC1 PASMCs under normoxia or hypoxia. *n* = 27–39 cells per group, scale bar:100 μm. **C** Western blot showing HIF1α levels in cells as in (**A**). *n* = 6 independent experiments per group. **D** PCNA protein level, **E** CCK8 assay, and **F** EDU assay in cells as in (**A**). *n* = 5 independent experiments per group for **D**, *n* = 6 independent experiments per group for **E**, and *n* = 4–7 independent experiments per group for **F**. Data are presented as mean ± SD. In **A**, **C** and **E**, *p* values were determined by one-way ANOVA followed by Bonferroni post hoc analysis. In **B**, **D** and **F**, *p* values were determined by Kruskal–Wallis test followed by Dunn’s post hoc analysis.

Reactive oxygen species (ROS) has been reported to regulates PASMC proliferation [26], so we hypothesize that ROS contributes to FUNDC1-induced PASMC proliferation through activating HIF1α. We firstly examined ROS production by ROS fluorescent probe 2’,7’-Dichlorofluorescin diacetate (DCFH-DA). Comparing with control cells, overexpression of FUNDC1 but not FUNDC1-ΔLIR caused largely increased ROS generation which could be inhibited by ROS scavenger N-Acetyl-L-cysteine (NAC), as indicated by the DCFH-DA fluorescent signal (Fig. 4A). Moreover, while hypoxia caused increased ROS production, it was markedly blocked by knockdown of FUNDC1 (Fig. 4B), suggesting that hypoxia-induced FUNDC1 activity increased ROS production in PASMCs.

Next, we examined the role of ROS in FUNDC1-mediated HIF1α activity and PASMC proliferation. NAC inhibited FUNDC1-upregulated HIF1α to the level equivalent to that in control cells (Fig. 4C). Likewise, NAC inhibited FUNDC1-induced increase of PCNA protein (Fig. 4D) and PASMC proliferation (Fig. 4E, F). Together, these results suggest that FUNDC1 increases ROS production which in turn upregulates HIF1α activity and subsequently results in PASMC proliferation during hypoxic PH.

### FUNDC1 mediates mouse hypoxic PH

**Fig. 5:**
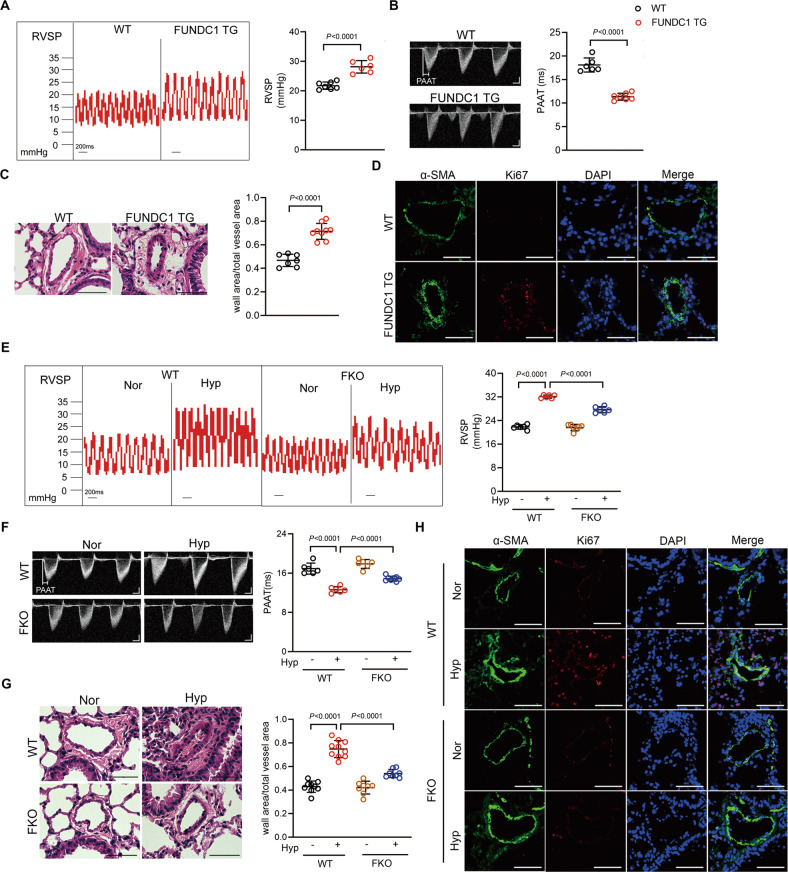
Increasing mitophagy results in mouse pulmonary hypertension (PH) and inhibition of mitophagy ameliorates mouse hypoxic PH. **A** Right ventricular systolic pressure (RVSP) and **B** pulmonary artery acceleration time (PAAT) of wild-type (WT) and *Fundc1* transgenic (FUNDC1 TG) mice. *n* = 6–7 mice per group. **C** The representative pulmonary artery images in the lung sections. *n* = 7–9 mice per group, scale bar: 50 μm. **D** Immunostaining of lung sections from WT and FUNDC1 TG mice with indicated antibodies. Green: α-SMA; red: Ki67; and blue: DAPI. scale bar: 50 μm. **E** RVSP and **F** PAAT of WT and *Fundc1* knockout (FKO) mice exposed to hypoxia for 3 weeks. *n* = 6–7 mice per group for **E**, and *n* = 6–8 mice per group for (**F**). **G** Pulmonary arterial wall thickness and **H** Ki67-positive cells in pulmonary arteries from WT and FKO mice under normoxia or hypoxia. *n* = 8–10 mice per group for **G**, scale bar: 50 μm. Data are presented as mean ± SD. Two-tailed unpaired Student’s *t* test and one-way ANOVA were used to compare two and multiple groups. Bonferroni post hoc analysis were carried out after ANOVA.

To further know whether FUNDC1 in vivo regulates hypoxic PH, we employed *Fundc1* transgenic mice (FUNDC1 TG) (Supplementary Fig. S4A, B) and *Fundc1* knockout mice (FKO) (Supplementary Fig. S4D) to establish gain-of- and loss-of-function models of mitophagy. The FUNDC1 TG/mt-Keima mice showed increased mitophagy comparing with WT/mt-Keima mice (Supplementary Fig. S4C). In contrast, the FKO/mt-Keima mice showed less mitophagy after hypoxia treatment as comparing with WT/mt-Keima mice after hypoxia (Supplementary Fig. S4E). Interestingly, at basal condition, the FUNDC1 TG mice displayed significantly higher RVSP values than WT mice (Fig. 5A). Echo-Doppler scans showed pulmonary artery acceleration time (PAAT) was shorter in FUNDC1 TG mice than in WT mice (Fig. 5B). In addition, right ventricular mass, calculated as right ventricle/(left ventricle+septum) (RV/(LV + S)), showed no difference between FUNDC1 TG and WT mice (Supplementary Fig. S5A). Moreover, FUNDC1 TG mice showed increased media wall thickness and increased Ki67-positive PASMCs, the proliferating marker, in distal pulmonary arteries (Fig. 5C, D), indicating that mitophagy causes PASMC proliferation and pulmonary vascular remodeling. Similar with our finding in FUNDC1 overexpressing PASMCs, FUNDC1 TG mice also showed more apoptotic resistance than WT mice as showed by TUNEL-positive cells in distal pulmonary arteries (Supplementary Fig. S6A). Thus, our results suggest that increasing FUNDC1 per se causes pulmonary vascular remodeling and pulmonary vascular hemodynamic changes of PH.

In contrast with FUNDC1 TG mice, the RVSP and PAAT of FKO mice had no difference with WT mice under normoxia condition (Fig. 5E, F). However, FKO mice showed markedly lower RVSP and higher PAAT than WT mice in response to hypoxia treatment for 3 weeks (Fig. 5E, F). FKO and WT mice showed no difference in RV/(LV + S) under normoxia condition, and similarly increased in response to hypoxia (Supplementary Fig. S5B). Moreover, FKO mice showed thinner pulmonary arterial wall thickness and less proliferation-positive PASMCs than WT mice in response to hypoxia (Fig. 5G, H). And distal pulmonary arteries from FKO mice had more TUNEL-positive cells than WT in response to hypoxia (Supplementary Fig. S6B), indicating that deletion of FUNDC1 ameliorates hypoxia-induced hemodynamics changes and vascular remodeling during PH. Collectively, these results suggest that FUNDC1 mediates hypoxic PH.

### Therapeutic implications of hypoxic PH by inhibition of FUNDC1-mediated mitophagy

**Fig. 6:**
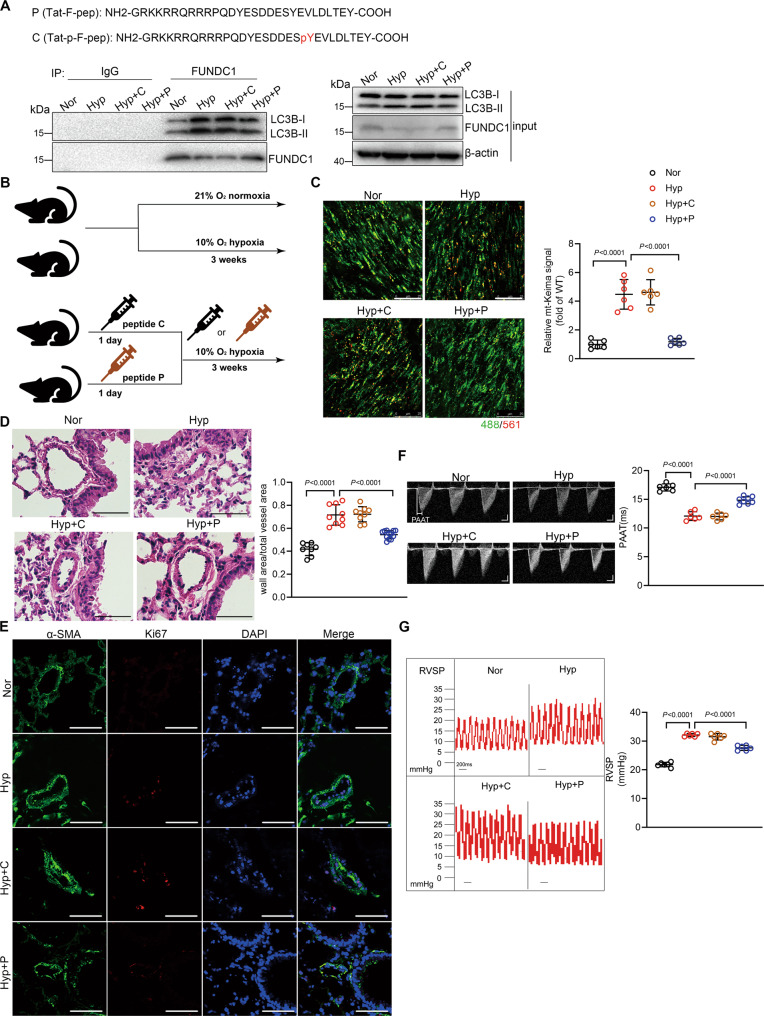
Specific inhibition of mitophagy attenuates hypoxic pulmonary hypertension (PH). **A** Cell-permeable peptide P (unphosphorylated FUNDC1 Tyr18) and C (phosphorylated FUNDC1 Tyr18). Co-IP showing the FUNDC1/LC3 interaction in pulmonary artery smooth muscle cells (PASMCs) pretreated with peptide P or C (250 nM) 1 h before hypoxia. **B** Timetable and procedure of hypoxia treatment with peptides. **C** Confocal images showing mt-Keima signal in mt-Keima mice treated as in (**A**). *n* = 4–6 mice per group, scale bar: 25 μm. **D** Pulmonary arterial wall thickness and **E** Ki67-positive cells in pulmonary arteries. *n* = 8–12 mice per group, scale bar: 50 μm. **F** RVSP and **G** PAAT. *n* = 6 mice per group for (**F**), and *n* = 6-7 mice per group for **G**. Data are presented as mean ± SD. *p* values were determined by one-way ANOVA. Bonferroni post hoc analysis were carried out after ANOVA.

PH is a severe disease causing heavy burden on patients’ life and even leading to death. However, current therapeutic interventions can only slow the progression but are unable to interrupt the pathogenesis of PH. Although originally identified as a hypoxia-related mitophagy receptor, FUNDC1 has been reported to have multiple functions including regulating mitochondria dynamics and Ca^2+^ signal [27, 28]. To specifically inhibit FUNDC1-mediated mitophagy function, we used a synthetic cell-penetrating peptide containing unphosphorylated Tyr18 of FUNDC1 (P) (Fig. 6A) [29]. Pretreatment of peptide P markedly inhibited hypoxia-induced interaction between FUNDC1 and LC3B-II, comparing with the phosphorylated peptide C control (Fig. 6A). In addition, specific inhibition of mitophagy by peptide P suppressed FUNDC1 overexpression- and hypoxia-induced ROS production and HIF1α activity in PASMCs (Supplementary Fig. S7A–D). Furthermore, pretreatment of peptide P also blocked FUNDC1 overexpression- and hypoxia-induced PASMC proliferation (Supplementary Fig. S7E–J), confirming that specific inhibition of mitophagy by the means of synthetic cell-penetrating peptide effectively interrupts hypoxia-mediated PASMC proliferation.

We then intraperitoneally injected peptide P or C (1 mg/kg/day) to mice 24 h before exposure to hypoxia (Fig. 6B). Comparing with control mice, peptide P effectively inhibited hypoxia-induced mitophagy in pulmonary artery medium from mt-Keima mice (Fig. 6C). Peptide P injection suppressed vascular obstruction and PASMC proliferation, as indicated by the decreased medial thickness in small pulmonary arteries and the less Ki67-positive PASMCs (Fig. 6D, E). More importantly, peptide P injection largely reduced RVSP and increased PAAT in response to hypoxia (Fig. 6F, G). However, peptide P had no significant effect on RV/(LV + S) in hypoxic conditions (Supplementary Fig. S7K). Thus, our data suggest that in vivo inhibition of FUNDC1-mediated mitophagy by injection of peptide may be a promising therapeutic strategy for hypoxic PH. Taken together, our present study reveals that FUNDC1-mediated mitophagy is upregulated during hypoxia, which causes PASMC proliferation, pulmonary remodeling, and ultimately PH, through increasing ROS production and HIF1α activity.

## Discussion

The present study found that FUNDC1-mediated mitophagy was upregulated in hypoxic PASMCs and in pulmonary arteries of hypoxic PH. Upregulation of FUNDC1 induced PASMC proliferation, pulmonary vascular remodeling, and PH, while downregulation of FUNDC1 attenuates hypoxia-induced PASMC proliferation, pulmonary vascular remodeling, and PH. A large body of evidence from either animal experiments or patients indicates that the generalized autophagy is activated in PH [10, 11]. However, other studies show decreased autophagy in rat PH models [30]. Moreover, the function of autophagy in the development of PH is still controversial, making the role of autophagy in PH a highly complicated question [13, 14, 31, 32]. These conflicting results may be due to different experimental conditions and species, different types of PH, and even in different pulmonary vascular cells such as pulmonary arterial endothelial cells or PASMCs. As a specific type of selective autophagy, little is known about the role of mitophagy in the progression of PH. Thus, the present study provides solid evidence to show that mitophagy plays a maladaptive role during hypoxic PH. However, our study mainly focuses on PASMCs in hypoxia-induced pulmonary vascular remodeling, more studies with other cell types and PH are needed to make our conclusion more accurate.

We found that in PASMCs, upregulation of FUNDC1 induced cell proliferation through increasing ROS production and HIF1α stabilization, and downregulation of FUNDC1 suppressed hypoxia-induced ROS production, HIF1α activity, and PASMC proliferation. Previous studies have reported that HIF1α plays a pivotal role in regulating PASMC proliferation especially during hypoxic PH [33], supporting our finding that FUNDC1-mediated HIF1α activity is responsible to PASMC proliferation. Excessive ROS production has been indicated in mitophagy [34]. Indeed, we found that upregulation of FUNDC1 causes increased ROS production. Further, our findings revealed that hypoxia-induced increase of FUNDC1 activity mediates HIF1α activity and PASMC proliferation through increasing ROS production. In consistent with our findings, there are studies showing that excessive ROS production participates in regulating hypoxia-induced PASMC proliferation and pulmonary vascular remodeling, and inhibition of ROS production reverses proliferation [35]. There are also studies demonstrating that ROS activates HIF1α [26], furthering supporting our finding that hypoxia-induced mitophagy mediates PASMC proliferation through ROS-HIF1α pathway. Interestingly, it has been reported that ROS also regulates mitophagy [36], pointing out a mutual regulation between ROS production and mitophagy which needs to be tested in future study in our experimental system. In addition, how FUNDC1-mediated mitophagy induces ROS production and activates HIF1α is not clear and merits further investigation.

The present study takes advantage of hypoxic mitophagy receptor FUNDC1 to investigate mitophagy activity in PASMCs and in mice. Combined with mt-Keima in PASMCs and in mice, we directly visualized mitophagy activity in PASMCs with FUNDC1 overexpression or knockdown, or in pulmonary arteries from FUNDC1 TG and FKO mice. And we found that mice with increased FUNDC1-mediated mitophagy activity per se displayed hypoxic PH phenotypes, and inhibition of FUNDC1-mediated mitophagy genetically or pharmacologically protects mice against hypoxic PH. Nevertheless, in addition of FUNDC1, there are several other mitophagy receptors such as BNIP3, NIX, and BCL2L13, and mitophagy receptor-independent pathways such as PINK-Parkin pathway which mediate mitophagy in most types of cells. It cannot be excluded that other mitophagy receptors or molecules provide compensatory functions in our experimental conditions. Further studies may be required to clarify this question.

Current therapeutic interventions of PH are not satisfactory on interrupting pulmonary vascular remodeling. In the present study, we explore the potential therapeutic strategy to intervene pulmonary vascular remodeling of hypoxic PH by targeting mitophagy. We found that intraperitoneal injection of a synthetic cell-penetrating peptide to specifically block FUNDC1-mediated mitophagy effectively inhibits hypoxic PASMC proliferation, pulmonary vascular remodeling, and PH. However, inhibition of mitophagy in our experimental conditions did not ameliorate RV hypertrophy. This may be because that our gain-of- and loss-of-function mitophagic models are not specific targeted on PASMCs and that hypoxia-induced signals in multi-organs which cause PH independent RV hypertrophy. Moreover, the synthetic peptide P is designed with the unphosphorylated Tyr18 of FUNDC1 to neutralize FUNDC1 activation. However, FUNDC1 Ser13 and Ser17 residues have also been reported to regulate mitophagy activity through post-translational modifications [18, 20]. Although the present study further performed FUNDC1 mutant with LIR motif deletion (FUNDC1-ΔLIR) to clarify the role of FUNDC1-mediated mitophagy in PASMCs, Ser13 and Ser17 residues are not in the LIR motif. Thus, developing new tools to precisely regulate mitophagy especially in PASMCs is needed for further studies and therapeutic interventions of PH.

In conclusion, the present study reveals the role of FUNDC1-mediated mitophagy in the pathogenesis of hypoxic PH. Mitophagy is upregulated in lung tissues in hypoxic PH, the upregulated mitophagy by FUNDC1 causes PASMC proliferation through ROS-HIF1α pathway, leading to pulmonary vascular remodeling, and hypoxic PH. Inhibition of FUNDC1-mediated mitophagy genetically or pharmacologically ameliorates hypoxic PASMC proliferation, pulmonary vascular remodeling, and PH, thus providing a therapeutic target for hypoxic PH.

## Materials and methods

### Reagents and antibodies

The following antibodies were employed in western blot: anti-TIM23 (1:1000; BD Biosciences, 611223), anti-LC3B (1:1000; Proteintech, 146000-1-AP), anti-PCNA (1:1000; Proteintech, 10205-2-AP), anti-β-actin (1:5000; Proteintech, 60008-1-1g) and anti-HIF1α (1:1000; Abclonal, A11945). Anti-p-18-FUNDC1 (1:500) and anti-FUNDC1 (1: 1000) polyclonal antibodies were produced by immunizing rabbits with synthesized, purified phosphorylated and non-phosphorylated peptides from FUNDC1 (Abgent, SuZhou, China). For immunofluorescence, the following antibodies were used: anti-Ki67 (1:100; Affinity, AF0198) and anti-α-SMA (1:200; Santa Cruz, sc-32251). The secondary antibodies used for immunofluorescence were: goat anti-mouse IgG Alexa Fluor-488 (1:200; Molecular Probes, A11029) and goat anti-rabbit IgG Alexa Fluor-561 (1:200; Molecular Probes, A11008). The cell-penetrating peptides P (NH2-GRKKRRQRRRPQDYESDDESYEVLDLTEY-COOH) and C (NH2-GRKKRRQRRRPQDYESDDESpYEVLDLTEY-COOH) were synthesized by Nanjing Jietai Company (Nanjing, China). All the above peptides were HPLC purified and had a purity of greater than 98%. 2’,7’-Dichlorofluorescin diacetate (DCFH-DA) was provided from Invitrogen (D2938), N-Acetyl-L-cysteine (NAC) from Beyotime Institute of Biotechnology (ST1546), chloroquine (C6628) and MG132 (M8699) from Sigma-Aldrich.

### Animals

All protocols of animal handling were approved by the Institutional Animal Care and Use Committee of Peking University Health Science Center, and conducted in accordance with National Institutes of Health Guide for the Care and Use of Laboratory Animals.

*Fundc1* knockout (FKO) mice, *Fundc1* transgenic (FUNDC1 TG) mice and mt-Keima mice were generated as previously described [29, 37].

### Hypoxic PH mouse model

8-week-old male C57BL/6J mice were exposed to 10% O_2_ a hypoxic chamber for 3 weeks to establish the hypoxic PH mouse models [38]. Age-matched wild-type (WT) mice were used as controls. Mice were randomly assigned to experimental groups, and the sample size were determined by power calculation. Mice were received 1 mg/kg cell-penetrating peptide P or C via intraperitoneal injection 1day before hypoxia exposure, and then every day during the hypoxia treatment period.

### Measurements of hemodynamics

Mice were lightly anesthetized with 3% isoflurane and maintained via a nose cone with 1.5% isoflurane (balanced with O_2_), images of pulmonary artery acceleration time (PAAT) were recorded by echocardiography Vevo 770 system (Visual Sonics). Right ventricular systolic pressure (RVSP) was measured by right heart catheterization using Millar pressure transducer catheter (size 1F). The catheter was inserted into the right jugular vein, advanced into superior vena cava, and finally into RV. After measurement of RVSP, the thorax was opened and the heart was dissected. And the weight ratio of the right ventricle (RV) divided by the sum of left ventricle (LV) and septum (S) (RV/(LV + S)) was determined as an index for RV hypertrophy.

### Construction of adenovirus

PCR production was cloned into pENTR™/TEV/D-TOPO vector (Invitrogen, MO, USA). The entry clone containing the DNA sequence of FUNDC1, myc-FUNDC1-ΔLIR or HIF1α was recombined into pAd/CMV/V5-DEST. The double-stranded oligonucleotide encoding sh-FUNDC1 or sh-HIF1α was cloned into pENTRTM/U6 vector and then recombined with the pAd/BLOCK-iT™ 6-DEST vector.

### Cell culture

Human embryonic kidney cell lines 293 A and 293 T purchased from the Institute of Biochemistry and Cell Biology of the Chinese Academy of Sciences (Shanghai, China) were mycoplasma free. All cells were authenticated by short tandem repeat (STR) DNA profiling. The rat primary culture of pulmonary artery smooth muscle cells (PASMCs) were prepared as previously described [39]. Cells were cultured with DMEM (Dulbecco’s modified eagle’s medium), which contained 10% fetal bovine serum, 1% penicillin and streptomycin in a humidified incubator with 5% CO_2_ at 37 °C. PASMCs were infected with adenovirus for 48 h in DMEM containing 10% fetal bovine serum as previously described [40]. PASMCs in hypoxic conditions were incubated with a gas mixture containing 1% O_2_, 5% CO_2_, and 94% N_2_.

### Mitochondrial isolation

Mitochondria were isolated from PASMC samples using differential centrifugation. Briefly, collected PASMCs in a pre-weighted falcon tube and kept the sample on ice at all time. And then centrifuged suspension twice at 600 × *g* for 15 min at 4 °C. The resulting supernatant was transferred to another clean tube, centrifuged at 8000 × *g* for 15 min at 4 °C and the resulting supernatant was discarded. The pellet was washed twice and centrifuged at 8000 × *g* for 10 min at 4 °C. Total protein was quantified by a BCA assay (Invitrogen, MO, USA).

### Western blot

Protein samples were subjected to SDS-PAGE and transferred to polyvinylidene fluoride (PVDF) membranes. The membranes were incubated overnight with indicated primary antibodies, followed by the appropriate HRP-conjugated secondary antibodies. Finally, immunoblots were evaluated with ChemiDoc XRS^+^ instrument (Bio-Rad).

### Co-Immunoprecipitation

Cells were collected and lysed in 1% NP-40 buffer (250 mM NaCl, 1% NP-40, 50 mM HEPES, 5 mM EDTA). Lysates were centrifuged for 10 min at 12,000 × *g*, and the supernatant was precleared with Protein A/G beads (Santa Cruz, CA, USA). After centrifugation, the precleared supernatant was incubated with indicated antibodies overnight at 4 °C. The following day, protein complexes were added with 50 μL of Protein A/G beads and incubated for 3 h. Finally, the collected protein complexes were washed 3 times and separated by 12% or 8% SDS-PAGE.

### Immunofluorescence

For immunofluorescence analysis, lung tissue samples were air-dried, permeabilized with PBS containing 0.3% Triton X-100 for 20 min, and processed for immunofluorescence staining as further described. Lung sections were stained with a mixture of anti-α-SMA and anti-Ki67 antibodies overnight at 4 °C. After the incubation, the slices were rinsed with PBS and incubated with goat anti-mouse IgG Alexa Fluor-488 for α-SMA and anti-rabbit IgG Alexa Fluor-561 for Ki67 for 2 h at room temperature. After rinsing, the sections were mounted using an anti-fading mounting medium and examined under the confocal microscope (TCS-SP8, Leica).

### Real-time PCR

The following primer pairs were used for quantitative real-time PCR: primers were performed as follows: *β-actin*, 5’- GAGACCTTCAACACCCCAGCC-3’(forward) and 5’-TCGGGGCATCGGAACCGCTCA-3’(reverse); *Vegf*, 5’- ATCCGCAGACGTGTAAATGTTCCT-3’(forward) and 5’- TCACCGCCTTGGCTTGTCAC-3’(reverse); *Glut1*, 5’- ACCTCAAATTTCATTGTGGG-3’(forward) and 5’- GAAGATGAAGAACCAGAACCAG-3’(reverse).

### CCK8 assay

CCK8 assay (Dojindo, Japan) was determined to detect cell proliferation. PASMCs were seeded into 96-well plates, and then incubated with 10 μL CCK8 solution for 2 h. Finally, absorbance was read at 450 nm using a spectrophotometer (Invitrogen, MO, USA).

### EDU assay

Proliferative cells were labeled with the Click-iT^TM^ EDU imaging kit (Life Technologies, Carlsbad, CA) according to the manufacturer’s instructions, and examined under the confocal microscope (TCS-SP8, Leica). The EDU incorporation rate was expressed as the ratio of EDU-positive cells to total DAPI-positive cells.

### Measurement of intracellular ROS

Intracellular ROS level was measured by oxidant-sensitive dyes DCFH-DA (Invitrogen, MO, USA). PASMCs were incubated with 10 μM DCFH-DA for 30 min in the dark at 37 °C. After washing thrice with PBS, the accumulation of ROS was visualized using confocal microscope (TCS-SP8, Leica).

### DNA constructs and production of lentivirus

The 20-nt target DNA sequences preceding a 5’-NGG PAM sequence at exon 2 in the genomic *Fundc1* locus (NC_005120.4) were selected for generating sing-guide RNA (sgRNA). The target sequence was 5’-CACCGATAGTAATGGGTGGCGTGAC-3’, and the control sgRNA sequence 5’-TGCGAATACGCCCACGCGATGGG-3’ was designed to target the *lacZ* gene from *Escherichia coli*. LentiCRISPRv2 and packaging plasmid psPAX2 were gifts from Zhang XZ (University of Chinese Academy of Sciences, China). To express SpGuides in the targeted cell, the oligos of top oligos 5’-CACCG-20nt and bottom oligos: 5’-AAAC-20nt-C (20nt: complimentary target *Fundc1* DNA sequence or *lacZ* sgRNA sequence) were annealed and cloned into the modified lentiCRISPRv2 by BsmBI (New England Biolabs, MA, USA). All clones were confirmed by DNA sequencing using a primer 5’-GGACTATCATATGCTTACCG-3’ from the sequence of U6 promoter that drives expression of sgRNAs. The successfully cloned sgRNA-lentiCRISPR vector, the packaging plasmid psPAX2, and the envelope plasmid VSV-G (Addgene: 8454) were mixed together and then added to a mixture of 6 μL lipofectamine 3000 (Thermo Fisher Scientific, MA, USA) in 90 μL OPTI-MEM (Thermo Fisher Scientific, MA, USA). Then the mixture was added to HEK 293 T cells for producing lentivirus for 4 days. The harvested lentivirus was used to infect PASMCs. Finally, the infected cells were selected in media containing 2 μg/mL puromycin (Sigma-Aldrich, MO, USA) and subjected to western blot analysis for confirmation of the protein expression of FUNDC1.

### Statistical analysis

All data are presented as means ± SD. Statistical analysis was performed with GraphPad Prism version 6.0 (GraphPad Prism Software, Inc, San Diego, CA) and the SPSS 24.0 (SPSS, Inc, Chicago, IL). Data groups (two groups) with normal distributions were compared using the two-tailed unpaired Student’s *t* test, and the Mann–Whitney *U*-test was used for variables without a normal distribution. Comparisons between multiple groups were assessed by one-way ANOVA followed by Bonferroni post hoc analysis to compare normally distributed continuous variables, non-normal distributed data were compared by Kruskal–Wallis test followed by Dunn’s post hoc analysis. *P* < 0.05 was considered statistically significant.

## Supplementary information

reproducibility checklist

Supplemental material

Original Data File

Original Data File

## Data Availability

All data and materials are available in the text and in supplementary information.
