# Supplementary material for: FUNDC1-mediated mitophagy and HIF1α activation drives pulmonary hypertension during hypoxia
Source: Cell Death Dis. 2022 Jul 21;13(7):634. doi: 10.1038/s41419-022-05091-2 (PMC9304375; doi:10.1038/s41419-022-05091-2)
Supplement: Supplementary file 2 — Supplemental material [file 41419_2022_5091_MOESM2_ESM.docx]

**Online Supplementary Methods**

***Transwell migration assay***

The transwell migration assay was performed using 24-well insert Transwell permeable supports containing a polycarbonate filter (Corning, Lowell, MA, USA). Briefly, cells were plated on the upper chambers, and conditioned medium was placed in the lower chambers. Cells were fixed with 4% formaldehyde and stained with HE. Nonmigrating cells on the upper side of the filters were removed.

***Flow cytometry***

To determine the apoptosis rate, PASMCs were washed with PBS and then incubated in a binding buffer containing Annexin V-fluorescein isothiocyanate and propidium iodide staining solution (BD Biosciences, USA). Finally, the detection of the proportion of cells in each period was carried out by flow cytometry (BD Biosciences, USA).

***TUNEL assay***

A TUNEL (Wanleibio, China) assay is commonly used for examining apoptosis. After TUNEL staining, lung sections were labeled with α-SMA and DAPI, and finally examined under the confocal microscope (TCS-SP8, Leica). The TUNEL-positive cells were defined as cells double labeled with TUNEL and DAPI.

***Statistical analysis***

Datasets were tested for normality of distribution with the Kolmogorov-Smirnov test. Data are presented as means ± SD. Differences between two groups were analysed using the two-tailed unpaired Student’s t-test for normal distribution data, or the Mann-Whitney U-test for non-normal distribution data. Differences between three or more groups were analysed using one-way ANOVA followed by Bonferroni post hoc analysis for variables with normal distribution or Kruskal-Wallis test followed by Dunn’s post hoc analysis for variables without normal distribution. Experimental replicate with an n<6 underwent non-parametric statistical evaluation. A *P* value <0.05 was considered to be statistically significant.

**Supplementary Figures**


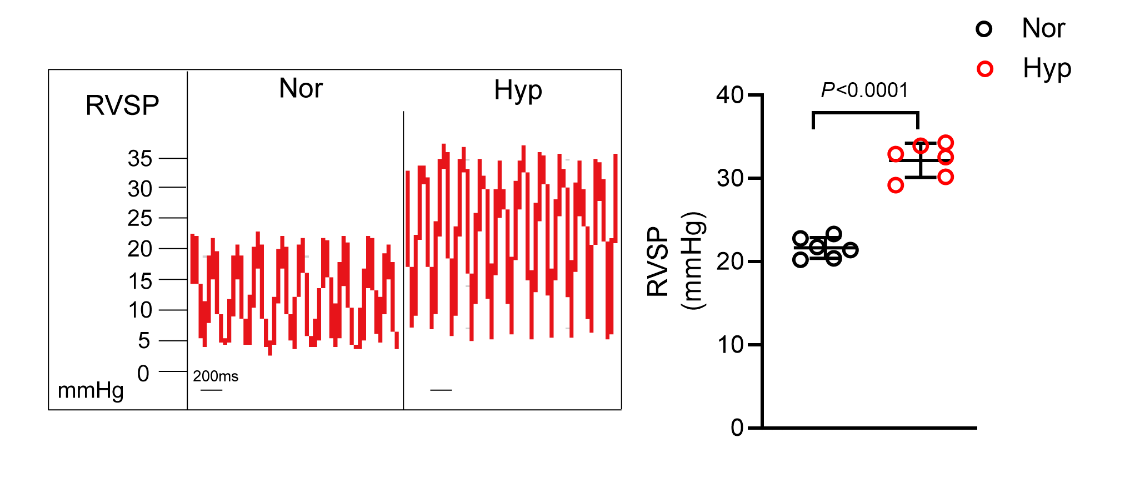


**Fig. S1. Hypoxia induces mouse pulmonary hypertension (PH).** Right ventricular systolic pressure (RVSP) measurement in mice after 3 weeks of normoxic (21% O_2_) or hypoxic mice (10% O_2_).n=6 mice per group. Data are presented as mean ± SD. p-values were determined by two-tailed unpaired Student’s t-test.


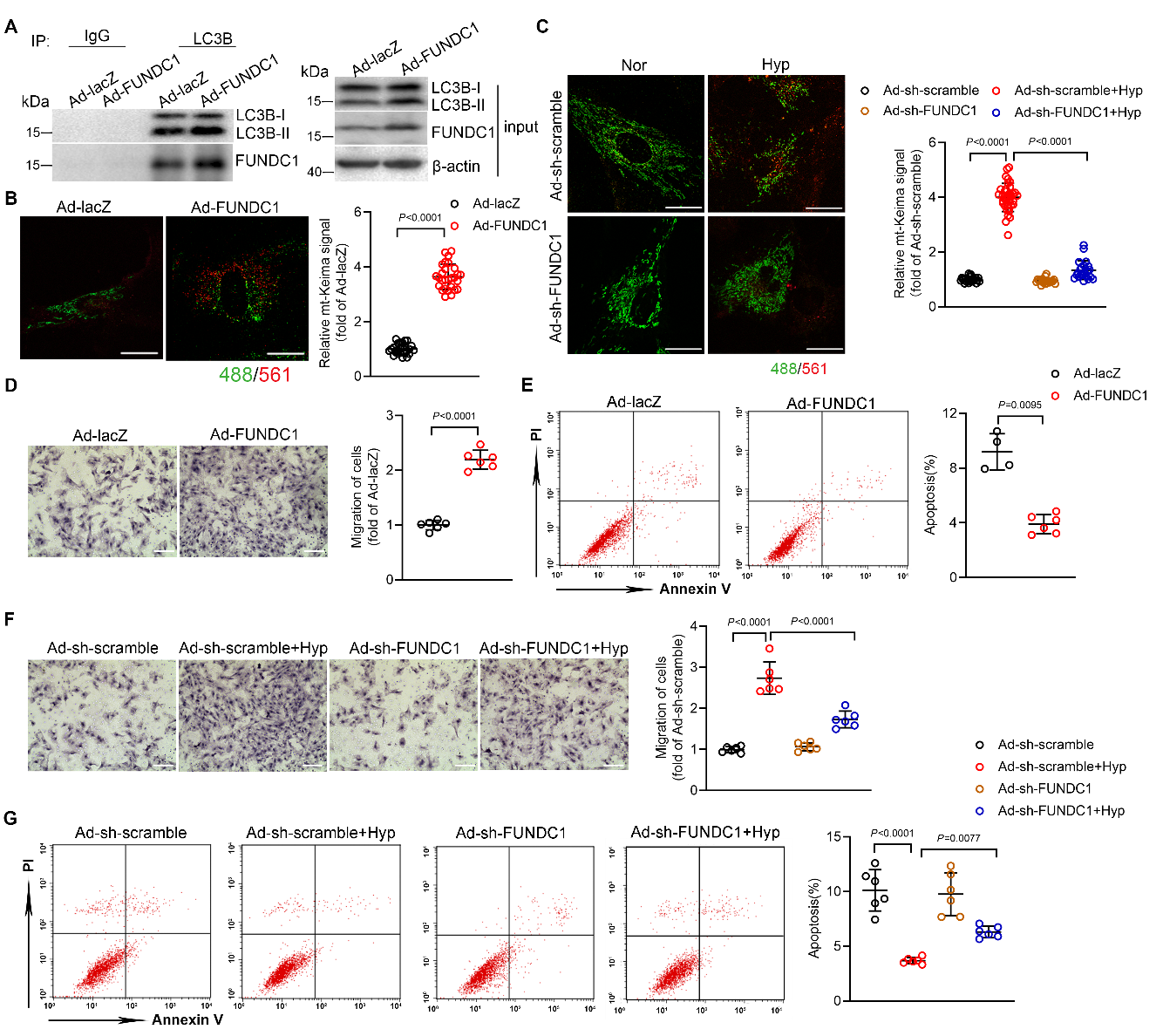


**Fig. S2. Mitophagy regulates pulmonary artery smooth muscle cell (PASMC) migration and anti-apoptosis in response to hypoxia. (A)** Co-IP of LC3B with FUNDC1 in Ad-lacZ or Ad-FUNDC1 PASMCs. **(B)** Representative confocal images showing mt-Keima fluorescent signal in mt-Keima-expressing PASMCs transfected with Ad-lacZ (n=26 cells) or Ad-FUNDC1 (n=28 cells). Scale bar: 25 μm. **(C)** Confocal images showing mt-Keima fluorescent signal in PASMCs transfected with Ad-sh-scramble or Ad-sh-FUNDC1 under normoxia or hypoxia for 24 h. n=19-36 cells per group. Scale bar: 25 μm. **(D)** Transwell assay showing migration of FUNDC1 expressing PASMCs. n=6 independent experiments per group. Scale bar: 50 μm. **(E)** Flow cytometry assay of cells as in (D). n=4-6 independent experiments per group. **(F)** Transwell assay of PASMCs transfected with Ad-sh-scramble or Ad-sh-FUNDC1 under normoxia or hypoxia for 24 h. n=6 independent experiments per group. Scale bar: 50 μm. **(G)** Flow cytometry assay of cells as in (F). n=6 independent experiments per group. Data are presented as mean ± SD. Two-tailed unpaired Student’s t-test and one-way ANOVA followed by Bonferroni post hoc analysis were used to compare two and multiple groups except for (E). In (E), data were analysed using Mann-Whitney U-test.


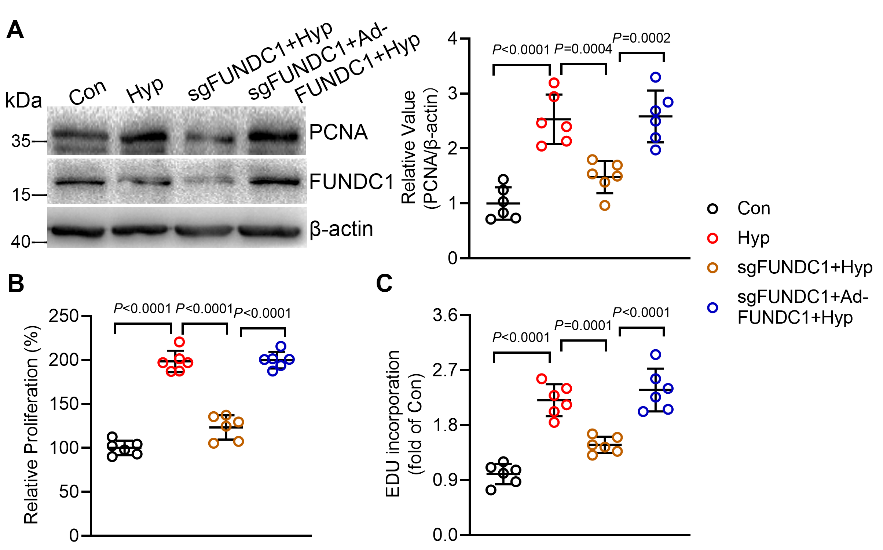


**Fig. S3. Re-expression of FUNDC1 abolishes FUNDC1 knockout-caused inhibition of pulmonary artery smooth muscle cell (PASMC) proliferation in response to hypoxia. (A)** Western blot showing PCNA levels in PASMCs by CRISPR/Cas-mediated knockout of FUNDC1 with or without expressing FUNDC1 under hypoxia. **(B)** CCK8 assay and **(C)** EDU assay of cells as in (A). n=6 independent experiments per group. Data are presented as mean ± SD, p-values were determined by one-way ANOVA followed by Bonferroni post hoc analysis.


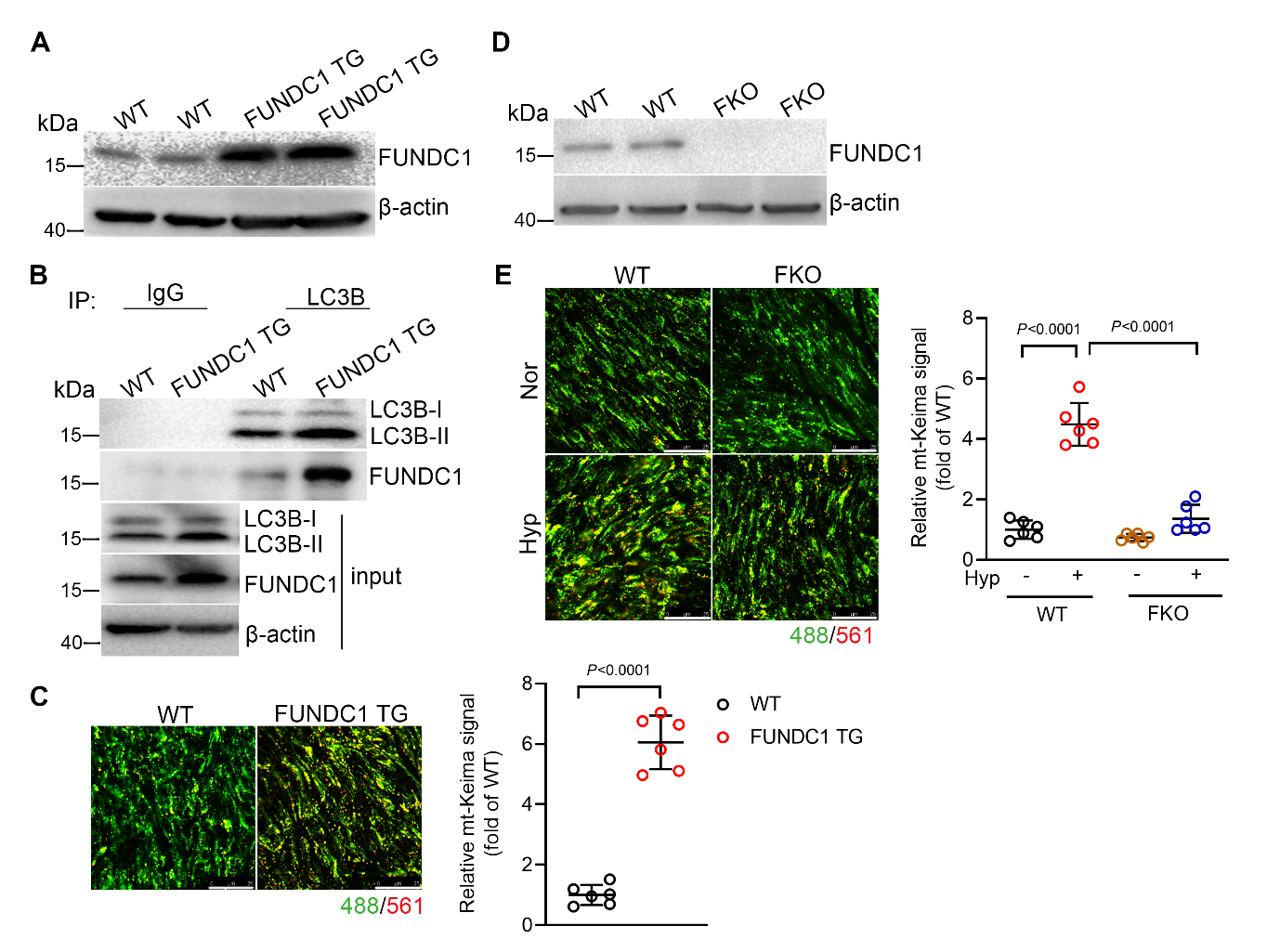


**Fig. S4. *Fundc1* transgenic (FUNDC1 TG) or *Fundc1* knockout (FKO) mice display increased or decreased mitophagy in pulmonary arteries *in vivo*. (A**) The protein levels of FUNDC1 in pulmonary arteries from wild-type (WT) and FUNDC1 TG mice. **(B)** Co-IP of LC3B with FUNDC1 in lung tissues from WT and FUNDC1 TG mice. **(C)** Confocal images of mt-Keima fluorescent signals in pulmonary arteries from WT/mt-Keima and FUNDC1 TG/mt-Keima mice. Quantification of mt-Keima signal is normalized to the WT control. n=6 mice per group. Scale bar: 25 μm. **(D)** The protein expression of FUNDC1 in pulmonary arteries from WT and FKO mice. **(E)** Confocal images of mt-Keima fluorescent signals in pulmonary arteries from WT/mt-Keima and FKO/mt-Keima mice under normoxia or hypoxia for 3 weeks. n=6 mice per group. Scale bar: 25 μm. Data are presented as mean ± SD. Two-tailed unpaired Student’s t-test and one-way ANOVA were used to compare two and multiple groups. Bonferroni post hoc analysis were carried out after ANOVA.


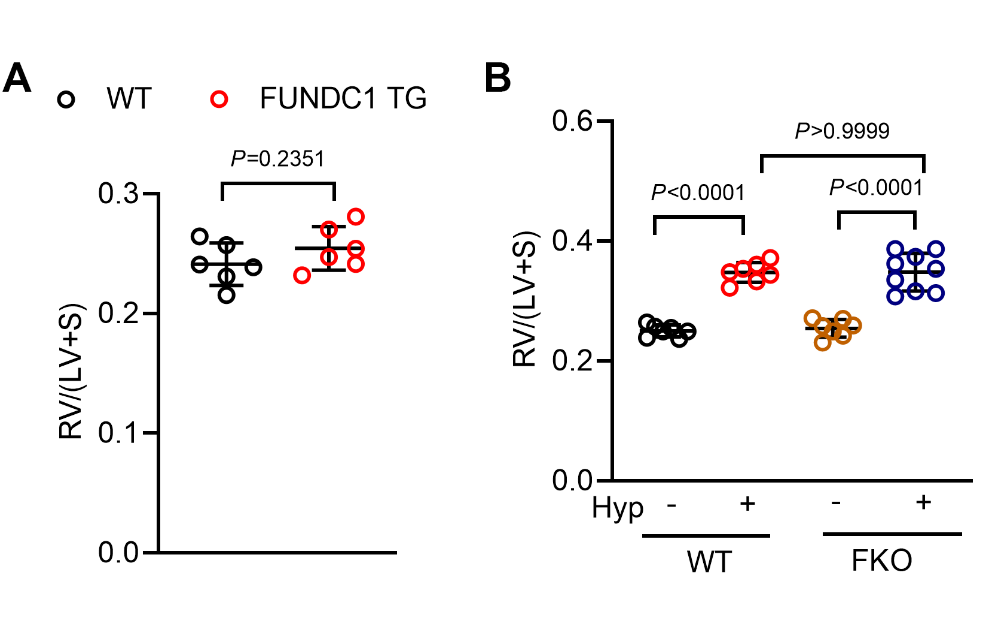


**Fig. S5. Mitophagy has no effects on right ventricular hypertrophy. (A**) Right ventricle/(left ventricle+septum) (RV/(LV+S)) in wild-type (WT) and *Fundc1* transgenic (FUNDC1 TG) mice. n=6 mice per group. **(B**) RV/(LV+S) in WT and *Fundc1* knockout (FKO) mice under normoxia or hypoxia. n=7-9 mice per group. Data are presented as mean ± SD. Two-tailed unpaired Student’s t-test and one-way ANOVA were used to compare two and multiple groups. Bonferroni post hoc analysis were carried out after ANOVA.


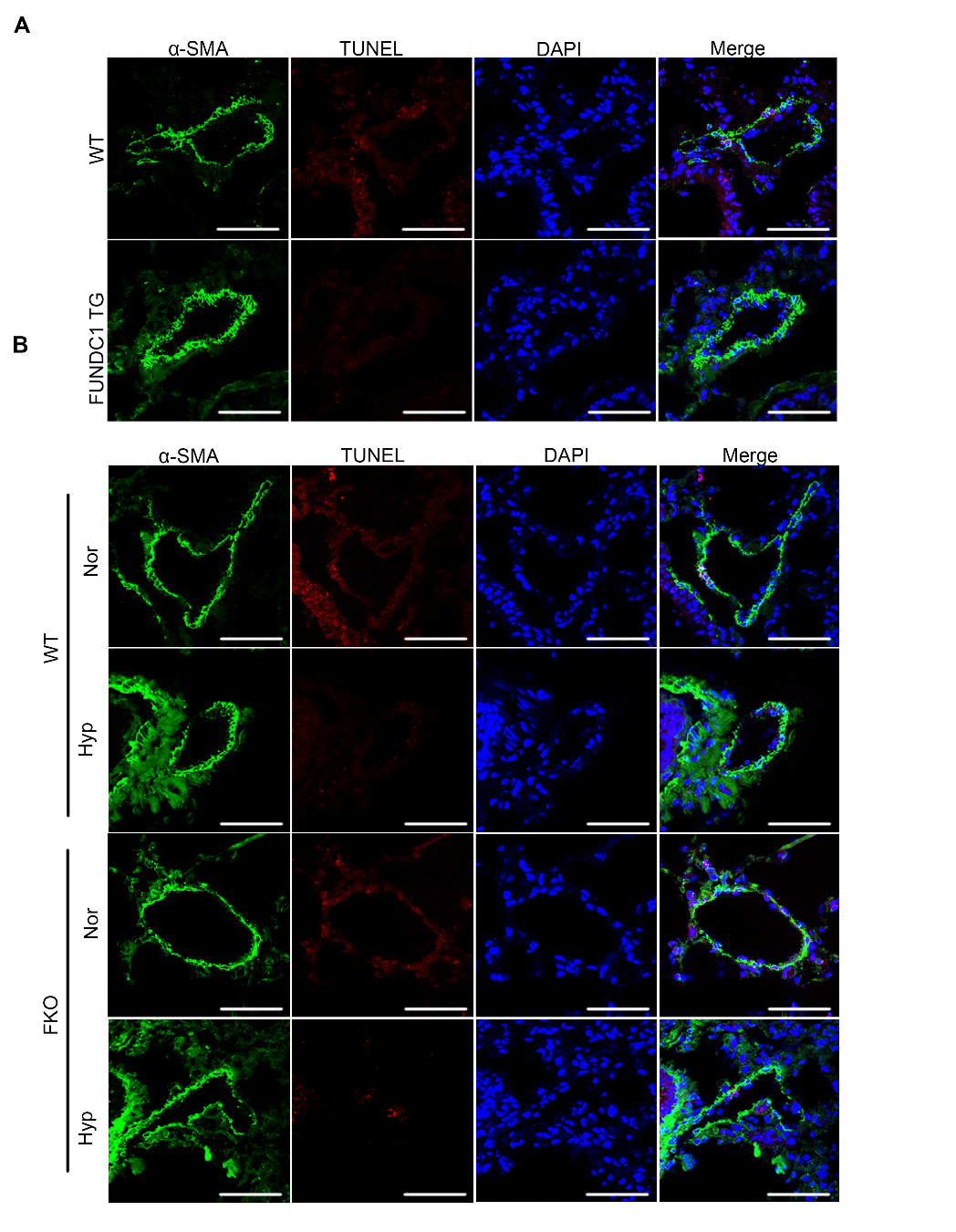


**Fig. S6. Apoptosis in the median of pulmonary arteries. (A)** TUNEL (red), α-SMA (green) and DAPI (blue) staining in pulmonary arteries from wild-type (WT) and *Fundc1* transgenic (FUNDC1 TG) mice. **(B)** TUNEL (red), α-SMA (green) and DAPI (blue) staining in pulmonary arteries from WT and *Fundc1* knockout (FKO) mice under normoxia or hypoxia. Scale bar: 50 μm.


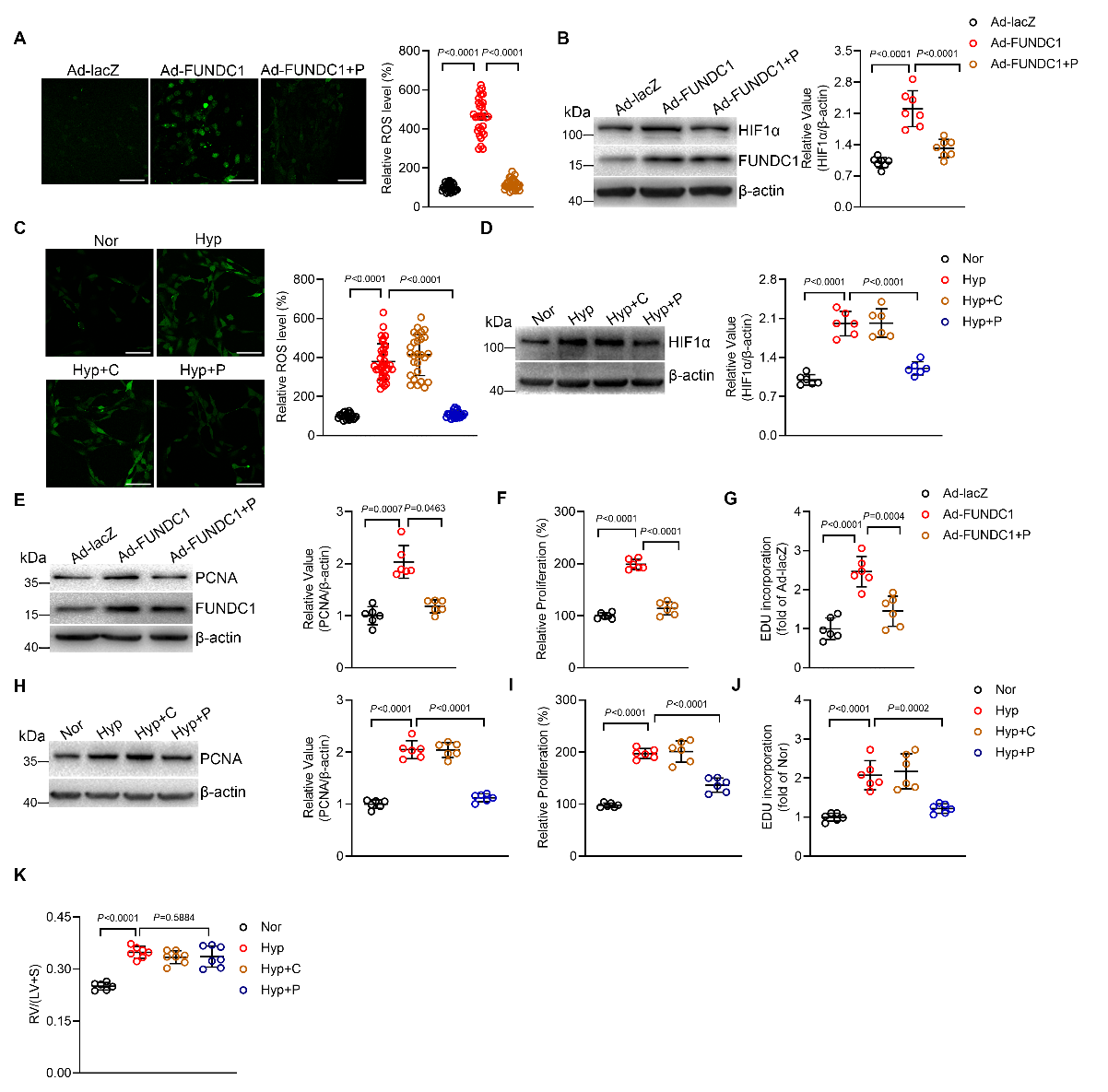


**Fig. S7. Inhibition of FUNDC1-mediated mitophagy by a cell-permeable peptide suppressed pulmonary artery smooth muscle cell (PASMC) proliferation. (A)** 2’,7’-Dichlorofluorescin diacetate (DCFH-DA) staining showing reactive oxygen species (ROS) production in Ad-FUNDC1 PASMCs with or without peptide P pretreatment. n=25-30 cells per group. Scale bar:100 μm. **(B)** Western blot showing hypoxia inducible factor 1α (HIF1α) protein levels in cells as (A). n=7 independent experiments per group. **(C)** DCFH-DA staining showing ROS production in PASMCs pretreated with peptide P under hypoxia. n=20-36 cells per group. Scale bar:100 μm. **(D)** HIF1α protein levels in cells as (C). n=6 independent experiments per group. **(E)** Proliferating cell nuclear antigen (PCNA) protein level, **(F)** CCK8 assay, and **(G)** EDU assay of PASMCs as (A). n=6 independent experiments per group for (E), (F) and (G). **(H)** PCNA protein level, **(I)** CCK8 assay, and **(J)** EDU assay of PASMCs as (C). n=6 independent experiments per group for (H), (I) and (J). **(K)** Right ventricle/(left ventricle+septum) (RV/(LV+S)) in wild-type (WT) mice injected with peptide P under hypoxia. n=6-7 mice per group. Data are presented as mean ± SD. p-values were determined by one-way ANOVA followed by Bonferroni post hoc analysis except for (E). In (E), the data were analysed using Kruskal-Wallis test followed by Dunn’s post hoc analysis.
