# Supplementary figures and images for: FUNDC1-mediated mitophagy and HIF1α activation drives pulmonary hypertension during hypoxia
Source: Cell Death Dis. 2022 Jul 21;13(7):634. doi: 10.1038/s41419-022-05091-2 (PMC9304375; doi:10.1038/s41419-022-05091-2)

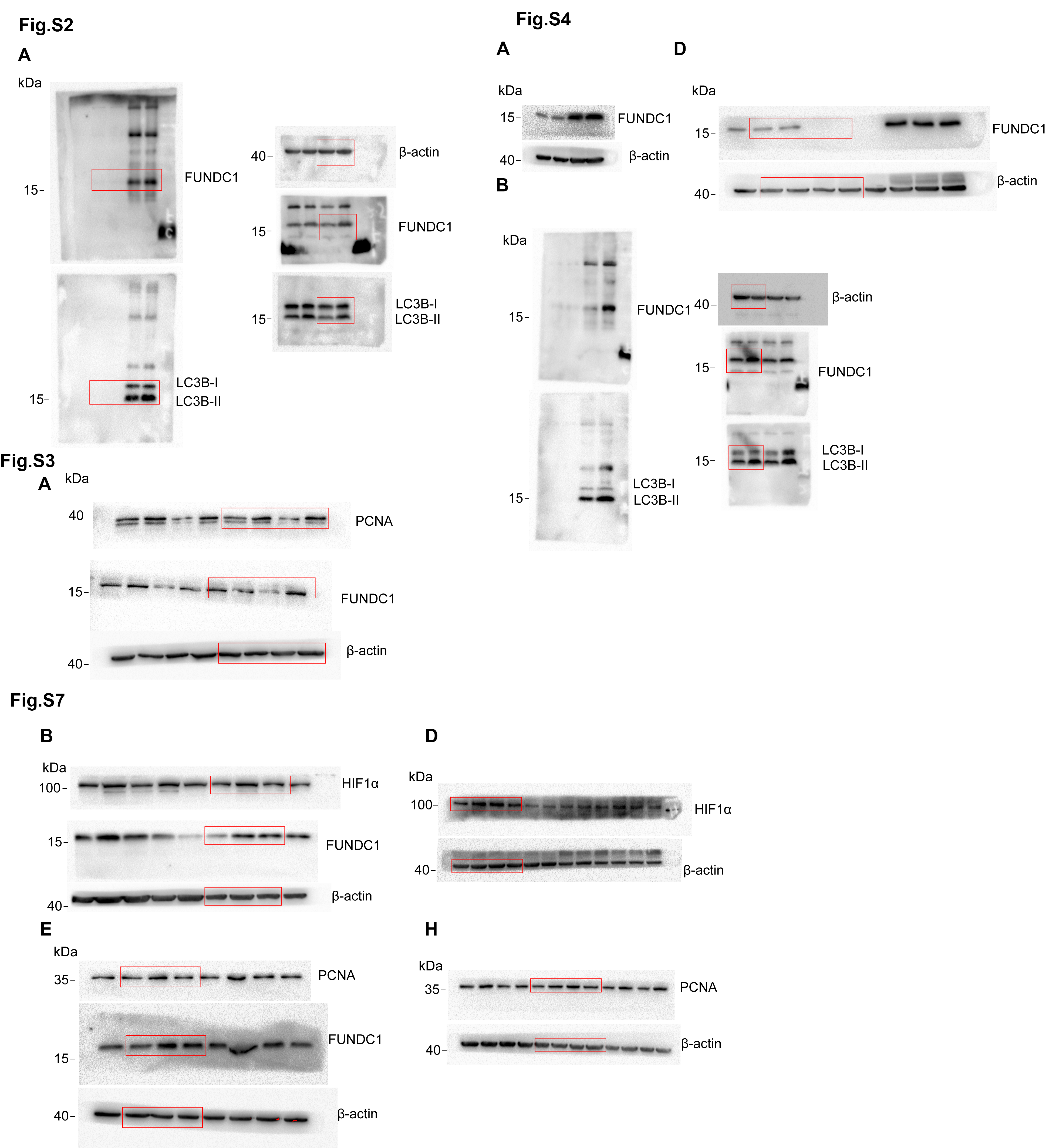

Supplement: Supplementary file 4 — Original Data File [file 41419_2022_5091_MOESM4_ESM.tif]
